# A novel modified-curcumin 2.24 suppresses MMPs in a periodontal-relevant cell culture model: a pilot study

**DOI:** 10.3389/froh.2026.1916570

**Published:** 2026-08-25

**Authors:** Isabella Ho, Cindy Leung, Nidhi Bhandari, Hsi-Ming Lee, Lorne M. Golub, Francis Johnson, Ying Gu

**Affiliations:** 1 Department of Oral Biology and Pathology, Stony Brook School of Dental Medicine, Stony Brook, NY, United States; 2 Department of Chemistry and Pharmacological Sciences, School of Medicine, Stony Brook University, Stony Brook, NY, United States; 3 Department of General Dentistry, Stony Brook School of Dental Medicine, Stony Brook, NY, United States

**Keywords:** CMC 2.24, gelatinase, collagenase, p38, THP-1

## Abstract

**Background:**

Matrix metalloproteinases (MMPs) play critical roles in periodontal tissue destruction. The present study evaluated the efficacy of a novel chemically modified curcumin 2.24 (CMC 2.24) in reducing MMP levels, activity, and its activation, in a periodontal relevant cell culture model involving human mononuclear THP-1 cells. In addition, the p38 mitogen-activated protein kinase (p38 MAPK) signaling pathway played a key role in the inflammatory process in response to various stimuli such as cytokines, bacterial antigens, and endotoxin lipopolysaccharide (LPS). Therefore, the effect of CMC 2.24 on p38 MAPK levels was evaluated as well.

**Methods:**

THP-1 monocytes were cultured and stimulated with LPS in the presence or absence of 5 µM CMC 2.24, Chemically modified tetracycline 3 (CMT-3), or doxycycline for 6 or 18 h. Conditioned media (CM) were analyzed for MMP-9 and collagenase levels using gelatin and collagen zymography. In addition, LPS-treated CM were incubated with 4-Aminophenylmercuric acetate (APMA) in the presence or absence of the above agents for an hour. MMP activation was evaluated by gelatin zymography. THP-1 MMP enzyme activity was analyzed using a fluorogenic substrate. p38 MAPK levels in THP-1 cell lysates were determined using the western blot technique.

**Results:**

CMC 2.24 significantly reduced elevated MMP-9 and collagenase levels compared to LPS controls. CMC 2.24 also inhibited APMA-induced activation of MMP-9 and reduced MMP enzymatic activity. Further, CMC 2.24 significantly suppressed p38 MAPK levels, whereas CMT-3 and doxycycline showed limited or no effect.

**Conclusions:**

CMC 2.24 functions as a highly pleiotropic inhibitor, acting at multiple regulatory levels: reducing MMP levels, activation, and enzymatic activity, while also downregulating MAPK signaling. These findings highlight CMC 2.24 as a promising therapeutic candidate for the modulation of inflammation-associated matrix degradation in chronic inflammatory diseases such as periodontitis.

## Introduction

Periodontitis is one of the most prevalent chronic inflammatory diseases worldwide, characterized by the progressive destruction of the tooth-supporting structures, including the periodontal ligament and alveolar bone (1–7). During the pathogenesis of periodontitis, anaerobic gram-negative bacteria and the lipopolysaccharides (LPS, endotoxin) in their cell walls stimulate innate and adaptive inflammatory immune responses. The body's immune response leads to an overproduction of inflammatory cytokines and matrix metalloproteinases (MMPs), such as MMP-9, MMP-1, MMP-8, and MMP-13 (8–14). MMPs actively participate in the breakdown of extracellular matrix components, accelerating tissue destruction (8, 9, 12, 13). MMP-9 is a key gelatinase that degrades connective tissue components, including gelatin and other proteins in the extracellular matrix (12, 15, 16). MMP-9 is synthesized as an inactive zymogen (92 kDa) and requires a disruption between the cysteine-zinc interaction (usually in the form of proteolytic cleavage) at its N-terminal pro-domain to become active (∼82 kDa) (12, 15, 16). Active MMP-9 has been directly linked to the destruction caused by periodontal disease (1, 2).

In addition to MMP-9, collagenases MMP-1, MMP-8, and MMP-13 are also linked to periodontal disease progression (8, 9, 12, 13, 17–19). MMP-1 (∼ 52 kDa), also known as collagenase-1, interstitial collagenase, or fibroblast collagenase, is primarily produced by fibroblasts, and to a lesser extent, macrophages, neutrophils, and endothelial cells (8, 9, 12, 13, 17). MMP-1 breaks down triple helical collagen Types I, II, and III by cleaving them into ¾ and ¼ fragments after unwinding them via interactions with collagen's binding domains. MMP-8, also referred to as neutrophil collagenase, or collagenase-2, is a collagenase (active ∼51 kDa, pro-form ∼85 kDa) common in neutrophils that primarily breaks down Type I collagen. MMP-8 is mainly produced by neutrophils but is also produced by activated macrophages, endothelial cells, chondrocytes, fibroblasts, and odontoblasts (8, 9, 12, 13, 17). Finally, MMP-13 (∼52–58 kDa), also known as collagenase-3, primarily breaks down Type II collagen, but can also degrade Type I collagen. MMP-13 is secreted by connective tissue cells like fibroblasts, chondrocytes (cartilage cells), and osteoblasts (8, 9, 12, 13, 17). Notably, collagen Type I is the most abundant in periodontal and gingival tissues (8, 9, 12, 13, 17).

Furthermore, the p38 mitogen-activated protein kinase (p38 MAPK) pathway is a critical signaling cascade activated in response to inflammatory stimuli such as periodontitis (9, 20). Activation of the p38 MAPK pathway upregulates the expression of various pro-inflammatory mediators, including MMPs, thereby amplifying tissue destruction during inflammatory diseases like periodontitis (9, 20, 21). Previous studies performed in our lab demonstrated that inhibition of the p38 MAPK pathway can significantly attenuate inflammatory responses in a natural periodontitis canine model.

Our laboratory has developed chemically modified tetracycline derivatives as MMP and inflammatory cytokine inhibitors. Low-dose doxycycline is a non-antimicrobial dosage of doxycycline hyclate (3, 22). Low-dose doxycycline was shown to be an effective inhibitor of matrix metalloproteinases (MMPs), while chemically modified tetracycline-3 (CMT-3) was further developed as a non-antibiotic anti-inflammatory agent, highlighting its therapeutic potential as a host-modulation strategy to target MMP activity (23–26).

Most recently, our laboratory has contributed to the development of a chemically-modified-curcumin 2.24 (CMC 2.24) as a newer generation MMP inhibitor. CMC 2.24 is a novel chemically modified tri-ketonic analog of curcumin with enhanced anti-inflammatory properties (1, 2, 7, 21, 27, 28). It resolves inflammation through a multi-target, host-modulatory approach (1, 2, 7, 21, 27). In previous studies, CMC 2.24 significantly reduced MMP and pro-inflammatory cytokine levels in a range of animal and tissue culture models of inflammatory diseases (1, 2, 21). CMC 2.24 was also found to reduce p38 MAPK levels in our animal model of periodontitis. However, its effects on MMP-9 (such as enzyme levels, enzyme activation and activity) and collagenases have not yet been fully characterized. Thus, in this study, we investigated the effects of CMC 2.24 on MMP-9, collagenases, and p38 MAPK levels using a periodontal-relevant cell line, the THP-1 cell line. THP-1 cells are a human monocytic cell line, and are frequently used in inflammation studies (29–32).

It should be noted that THP-1 cells can be differentiated from monocytes into macrophage-like cells through exposure to phorbol 12-myristate 13-acetate (PMA), also known as PMA induction (31). PMA-induced differentiation is accompanied by morphological and functional changes that can be evaluated by different methods, including flow cytometry (21). In general, macrophages can be categorized into two broad subsets, the M1 and M2 phenotypes. M1 is a pro-inflammatory phenotype, and M2 is a pro-resolving or anti-inflammatory phenotype (21, 31). Under chronic inflammation, macrophages will demonstrate the M1 phenotype. In contrast, under certain treatments that promote the conversion of M1 to M2, inflammation can be reduced. An earlier study conducted by our lab found that CMC2.24 treatment shifted AGE-challenged macrophages from a pro-inflammatory M1 phenotype toward a pro-resolving M2 phenotype, significantly increasing the M2/M1 ratio (21). Flow cytometry showed that CMC2.24 acted as a phenotypic “switch” by suppressing M1 polarization and promoting resolving M2 polarization (21).

THP-1 cells were used without differentiation by PMA. This was a deliberate choice to allow investigation of CMC 2.24's effects on early-stage, monocytic inflammatory responses rather than on macrophage-like activity, since previous studies already differentiated other cell lines into macrophages (21).

## Materials and methods

### Chemical reagents

CMC 2.24 was synthesized and provided by Chem-Master Intl., Inc. (Stony Brook, NY, USA; 99.5% pure). All other cell culture reagents and chemicals were obtained from Thermo Fisher Scientific (Waltham, MA, USA).

### Cell culture

The human mononuclear THP-1 cell line was purchased from the American Type Culture Collection (ATCC®, Manassas, VA, USA). Cells were cultured at a concentration of 1 × 10^6^ cells/mL in serum-free RPMI media for either 6 or 18 h in the absence or presence of 50 ng/mL *Escherichia coli* LPS (Sigma-Aldrich, St. Louis, MO, USA) In addition to LPS or vehicle, cells (*n* = 3) were incubated with 5 µM of CMC 2.24, CMT-3, or doxycycline respectively.

### Gelatin zymography for MMP-9 analysis

After 6 and 18 h of incubation respectively, THP-1 conditioned media (CM) were collected and analyzed for MMP-9 levels. 7.5% polyacrylamide was co-polymerized with 1 mg/mL gelatin (Thermo Fisher Scientific, Inc., Waltham, MA, USA), and samples were electrophoresed as described previously (1, 2). Gels were incubated with Novex™ Zymogram Renaturing Buffer (Thermo Fisher Scientific, Inc., Waltham, MA, USA) for an hour, and then overnight at 37°C with Novex™ Zymogram Developing Buffer. Gels were stained with Coomassie SimplyBlue SafeStain (Invitrogen Corp., Carlsbad, CA). Levels of MMP-9 were determined by scanning the lytic bands with Invitrogen™ iBright™ FL1000 Imaging Systems (Thermo Fisher Scientific, Inc., Boston, MA, USA), and quantified using ImageJ software.

To evaluate the effect on MMP-9 activation, after 18 h of incubation with LPS, the THP-1 CM were collected and incubated with 1 mM APMA for 1 h at 37°C in the presence of either 10 μM of CMC 2.24, CMT-3, or doxycycline. Gelatin zymography was used to analyze levels of pro (92 kDa) and active (82 kDa) MMP-9 in each sample. Levels of pro and activated MMP-9 were quantified as described above.

### Collagen zymography

After 6 or 18 h of incubation, the THP-1 CM were collected and analyzed for collagenase levels. 10% polyacrylamide was copolymerized with Type I rat tail collagen, with a final Type I collagen concentration of 0.3 mg/mL (33, 34). After electrophoresis, the gels were incubated in Novex™ Zymogram Renaturing Buffer for one hour and then at 37°C in Novex™ Zymogram Developing Buffer. After incubation, the gels were stained with Coomassie SimplyBlue SafeStain (Invitrogen Corp., Carlsbad, CA) and destained overnight with a 20% methanol/10% acetic acid solution. Collagenase levels were determined as described above.

### MMP enzyme activity assays

MMP Enzymatic activity was assessed using a fluorogenic collagenolytic-specific substrate, Mca-KPLGL-Dpa-AR-NH₂ (R&D Systems, Minneapolis, MN, USA). CM collected from THP-1 cells were incubated with 10 µM of fluorogenic substrate and 5 µM of CMC 2.24, CMT-3, doxycycline, or 1 mM 1,10-phenanthroline (positive control for MMP inhibition) for a duration of ten hours in the SpectraMax i3x microplate reader (Molecular Devices, San Jose, CA, USA). Fluorescence was read every 30 min in relative fluorescence units (RFUs). Fluorescence was measured at an excitation wavelength of 320 nm and an emission wavelength of 405 nm.

### Western blotting: p38 MAPK

After 18 h of incubation, THP-1 cells were lysed using a buffer composed of 1% SDS, 150 mM NaCl, 5 mM EDTA, 50 mM Tris-HCl, and 1% Triton X-100, supplemented with Halt™ Protease and Phosphatase Inhibitor Cocktail, EDTA-free (1:100 dilution; Thermo Fisher Scientific, Inc., Waltham, MA, USA). Lysates were centrifuged at 4,000 rpm for 20 min at 4°C to pellet cellular debris, and the supernatants were collected for protein analysis. Total protein concentration of THP-1 cell lysates was determined using the Bradford protein assay (Bio-Rad Laboratories, Hercules, CA, USA) and a bovine serum albumin standard curve (Thermo Fisher Scientific, Inc., Waltham, MA, USA). Before loading, samples (*n* = 3) were treated with 1% DTT loading buffer (Active Motif, Carlsbad, CA, USA) and boiled at 100°C for 2 min. The samples containing equal amounts of total protein were electrophoresed on SDS-PAGE (7.5% resolving and 4% stacking polyacrylamide gels) for 1.5 h at 120 V. Proteins were transferred to nitrocellulose membranes overnight at 25 V in 1X Towbin transfer buffer (Bio-Rad Laboratories, Hercules, CA, USA) containing 20% (v/v) methanol. Following transfer, membranes were washed three times in 1X Tris-buffered saline with 0.1% Tween 20 (TBS-T) for five minutes each. Membranes were then incubated overnight at 4°C with a primary antibody against p38 MAPK (1:1000 dilution; Cell Signaling Technology™, Danvers, MA, USA). After washing, membranes were incubated for two hours at room temperature with an HRP-conjugated goat anti-rabbit secondary antibody (1:5000 dilution; Cell Signaling Technology™, Danvers, MA, USA). Protein bands were visualized using SuperSignal™ West Dura chemiluminescence HRP substrate (Thermo Fisher Scientific™, Waltham, MA, USA) and detected using the Invitrogen™ iBright™ FL1000 Western Blot Imaging System (Thermo Fisher Scientific™, Waltham, MA, USA). Band intensities were quantified using ImageJ software.

### Statistical analysis

Student's *t*-test specifically comparing Cells + LPS (control) to treatment groups were used for statistical analysis. A *p*-value of less than 0.05 was considered statistically significant. Student t-tests were performed using Microsoft Excel (Microsoft Corp., Redmond, WA, USA). Additionally, one-way ANOVA was performed using JASP Software (University of Amsterdam., Amsterdam, Netherlands) with *p* < 0.05 as statistically significant.

## Results

### CMC 2.24 reduces MMP-9 Levels

**Figure 1:**
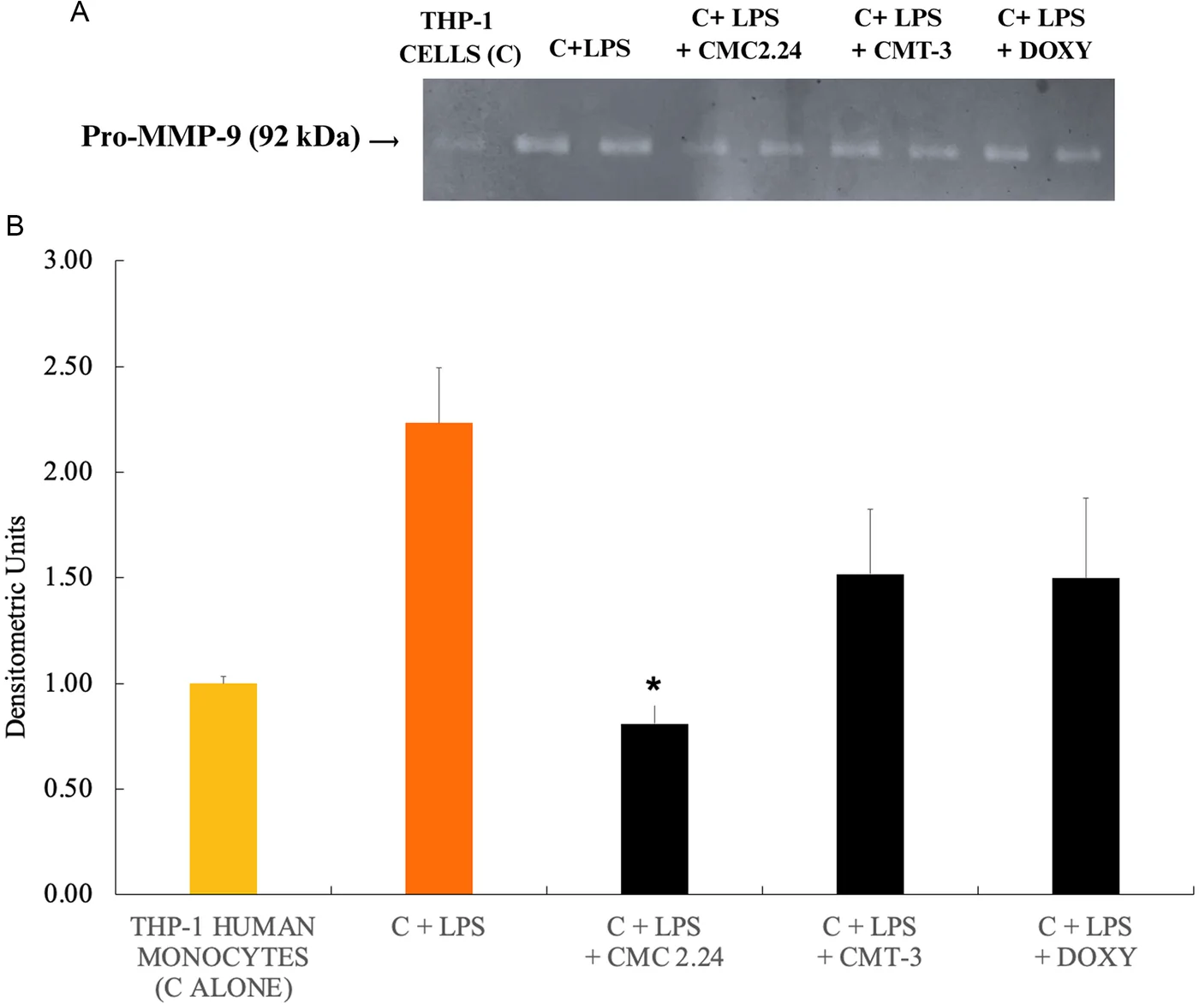
**(A)** MMP-9 levels in THP-1 CM At 6 hours (gelatin zymogram) THP-1 cells (1 × 10^6^ cells/ml) were cultured in serum-free media (37°C, 5% CO_2_/95%O_2_) with LPS (50 ng/mL) for 6 h with 5 µM of CMC 2.24, CMT-3, doxycycline, or vehicle alone. MMP-9 levels in the CM were analyzed by gelatin zymography. **(B)** MMP-9 Levels In THP-1 CM At 6 H (Quantified by ImageJ). Densitometric band intensity of MMP-9 levels (as shown in Figure 1A) were quantified using ImageJ. *: *p* < 0.05.

**Figure 2:**
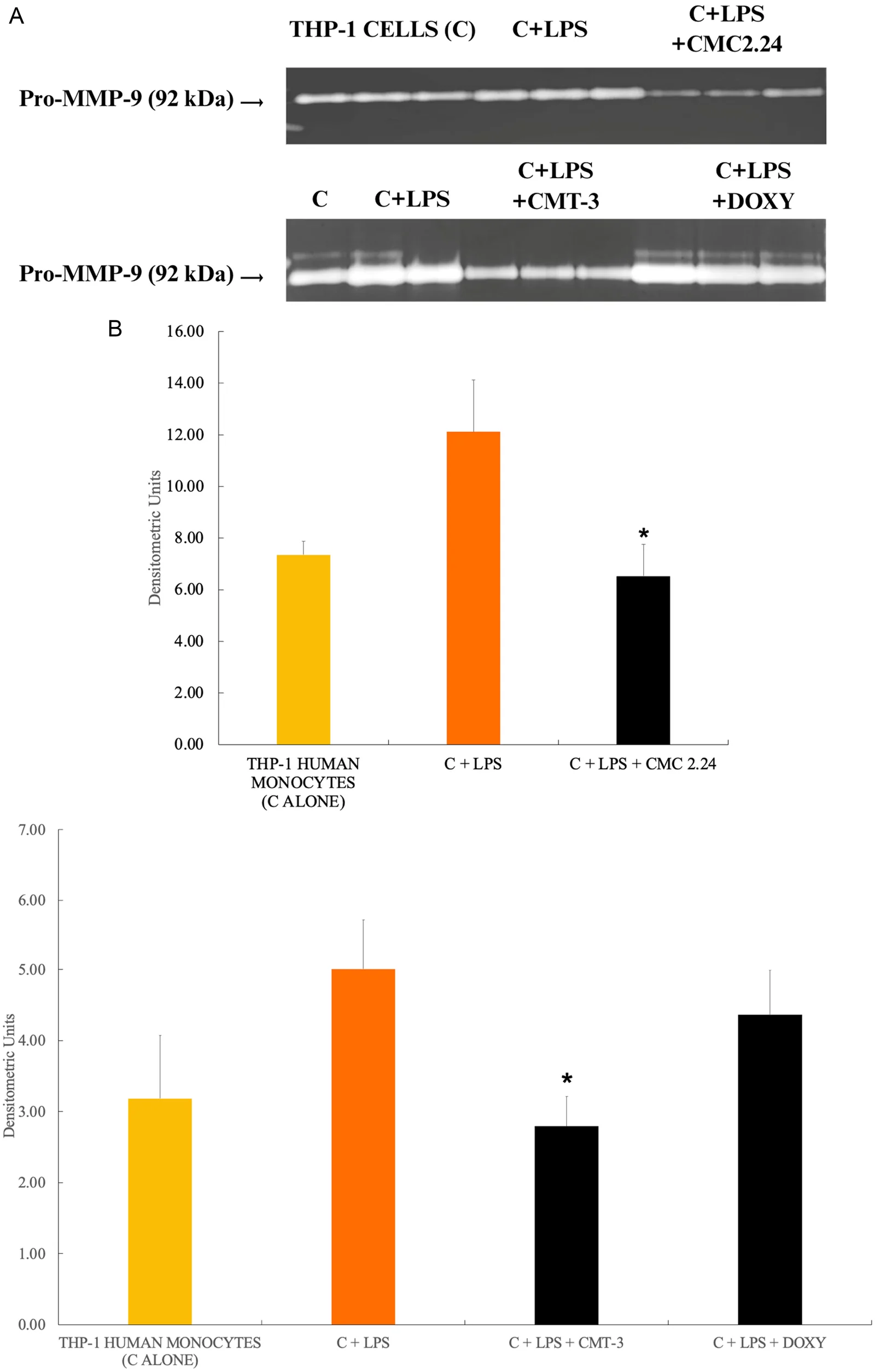
**(A)** MMP-9 levels in THP-1 CM At 18 h (gelatin zymogram). THP-1 cells (1 × 10^6^ cells/ml) were cultured in serum-free media (37°C, 5% CO_2_/95%O_2_) with LPS (50 ng/mL) for 18 h with 5 µM of CMC 2.24, CMT-3, doxycycline, or vehicle alone. MMP-9 levels in the CM were analyzed by gelatin zymography and quantified using ImageJ. *: *p* < 0.05. **(B)** MMP-9 Levels In THP-1 CM At 18 H (Quantified by ImageJ). Densitometric band intensity of MMP-9 levels (as shown in Figure 2A) were quantified using ImageJ. *: *p* < 0.05.

To determine the effects of CMC 2.24 on MMP-9 levels, gelatin zymography was performed on THP-1 cells after both 6 (Figure 1A) and 18 (Figure 2A) hours of incubation. Gelatin zymography results showed that pro-MMP-9 levels in LPS-treated THP-1 cell CM was significantly increased compared to vehicle alone by 123.18% at 6 h (Figure 2A) and by 61.01% at 18 h (Figure 2B).

Pro-MMP-9 levels were significantly inhibited compared to the cells + LPS control at 6 h by CMC 2.24 (63.02%, *p* < 0.01). CMT-3 inhibited Pro-MMP-9 levels by 31.94% and doxycycline inhibited Pro-MMP-9 levels by 34.34% but did not produce a significant effect. A one-way ANOVA revealed a significant effect of treatment on MMP-9 levels (p).

At 18 h, Pro-MMP-9 levels were significantly inhibited by CMC 2.24 (46.3%, *p* < 0.05) and CMT-3 (44.2%, *p* < 0.05). Doxycycline did not produce a significant effect. Additionally, a one-way ANOVA revealed a significant overall effect of treatment on pro-MMP-9 levels at 18 h, (*p* = 0.001).

### CMC 2.24 reduces activation of MMP-9

**Figure 3:**
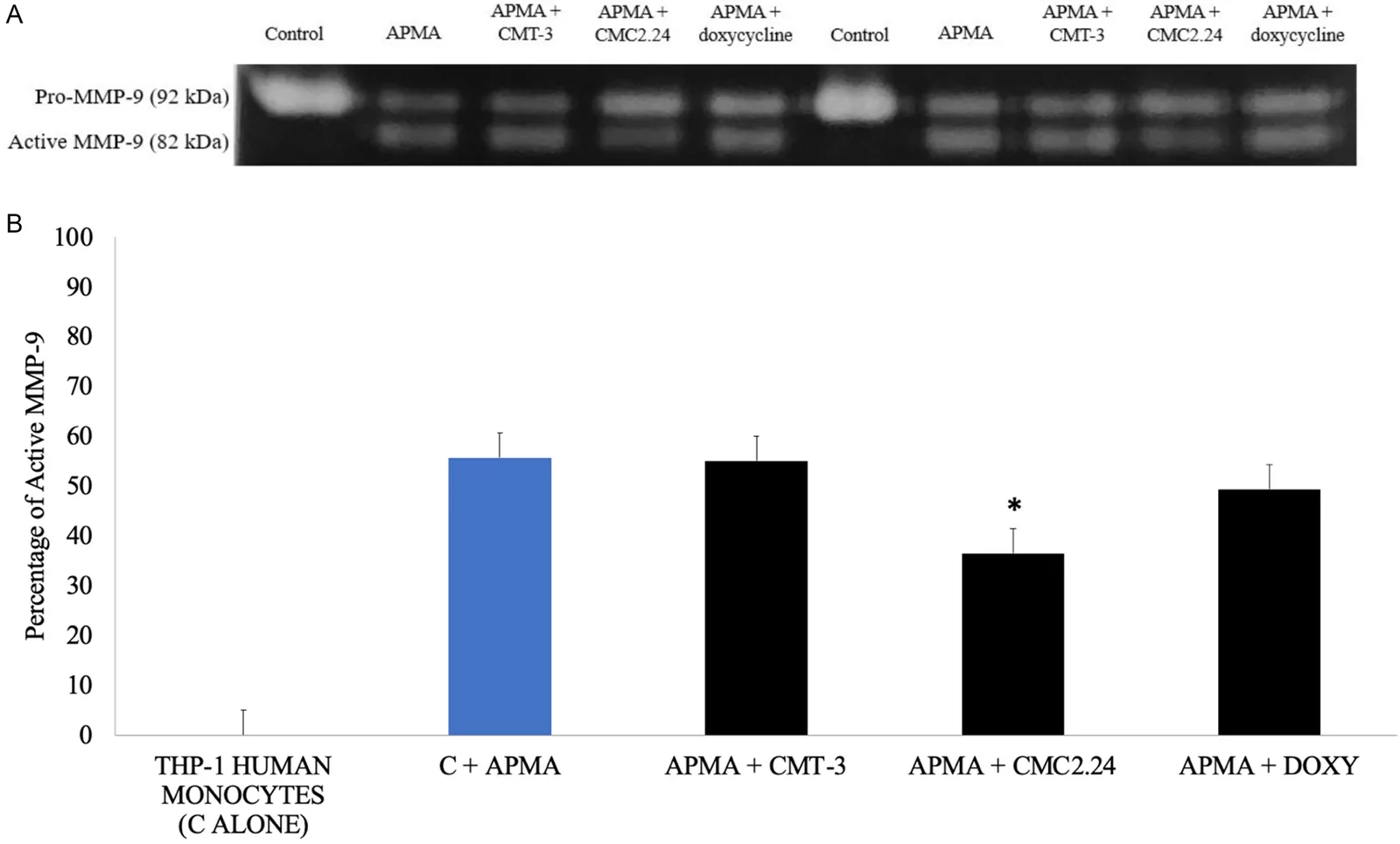
**(A)** MMP-9 activation in APMA activated LPS-stimulated THP-1 CM (gelatin zymogram). THP-1 cells (1 × 10^6^ cells/ml) were cultured in serum-free media (37°C, 5% CO_2_/95%O_2_, 18 h) with LPS (50 ng/mL). CM were collected and then co-incubated with 1 mM APMA and 10 µM of CMC 2.24, CMT-3, doxycycline, or vehicle alone for 1 h at 37°C. **(B)** MMP-9 Activation In APMA Activated LPS-Stimulated THP-1 CM (Quantified by ImageJ). Densitometric band intensity of pro and active MMP-9 levels in Figure 3A were analyzed and quantified using ImageJ. Percent of active MMP-9 was calculated. *: *p* < 0.05.

Gelatin zymography analysis (Figure 3A) showed that MMP-9 was activated by APMA by an average of 55.61%. 10μM of CMC2.24 showed an average activation of 36.36%, with a 34.49% reduction in MMP-9 activation (*p* < 0.05) (Figure 3B). 10μM of CMT-3 and doxycycline both did not show any significant effect on MMP-9 activation. Furthermore, one-way ANOVA showed a significant overall effect of treatment (*p* < 0.001).

### CMC 2.24 reduces collagenase levels

**Figure 4:**
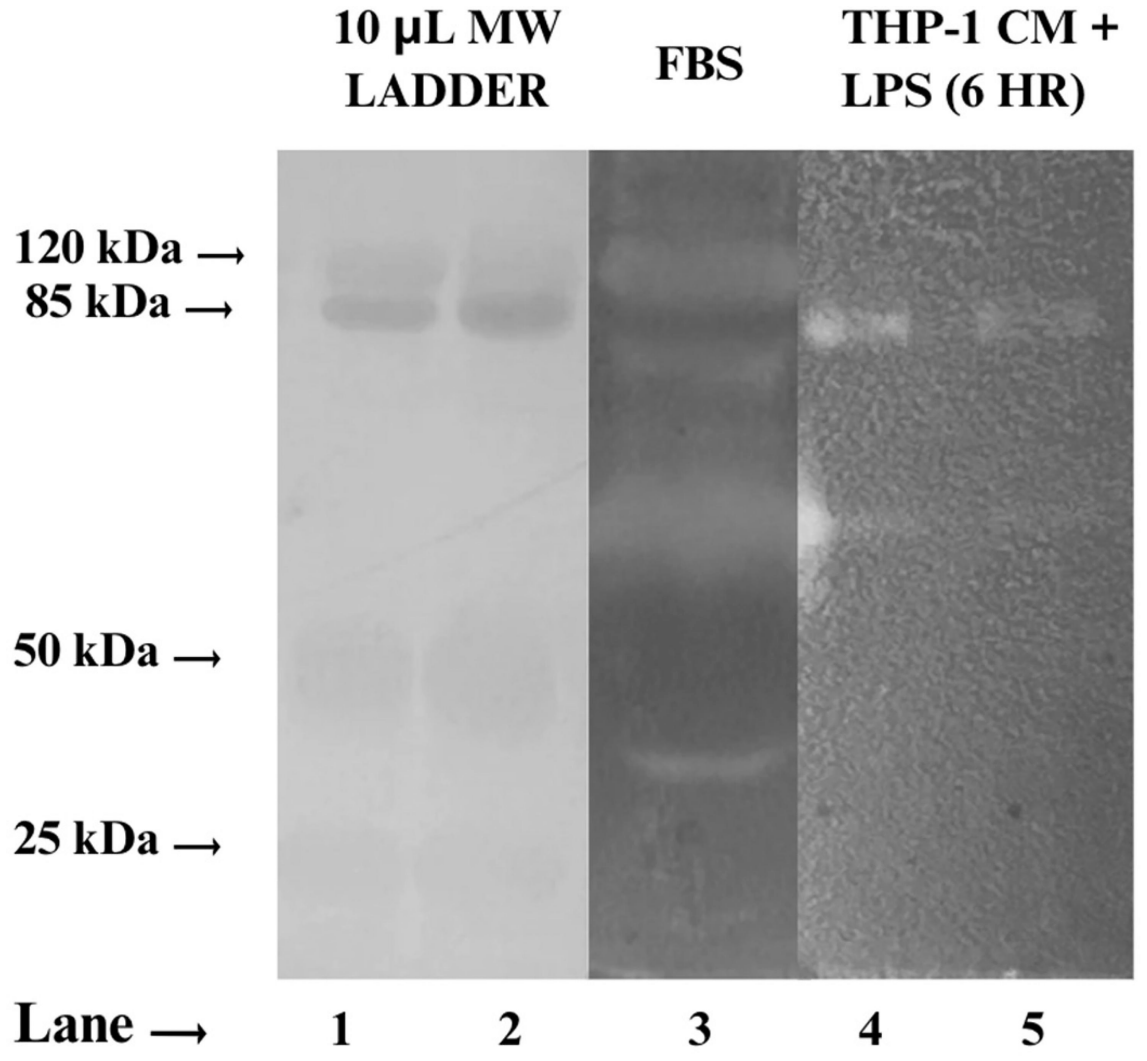
Molecular weight identification of collagenolytic band detected in THP-1 CM (collagen zymogram). A pre-stained molecular weight ladder (lanes 1-2) was used to estimate the molecular weight of the collagenase in THP-1 CM. Fetal bovine serum (FBS) was used as a positive control for collagenase activity (lane 3). THP-1 CM was treated with LPS for 6 h (lanes 4-5) and electrophoresed on a collagen-containing gel (0.3 mg/mL). After electrophoresis, the portion of the gel containing CM samples was developed to visualize the collagenase levels. Band intensities were quantified by densitometric analysis using ImageJ.

Collagen zymography using rat tail Type I collagen (0.3 mg/mL) was performed to evaluate the effects of the inhibitors on collagenase levels in THP-1 CM. A pre-stained molecular weight ladder (Figure 4) indicated a predominant band at approximately 85 kDa, consistent with the molecular weight of pro-MMP-8. Other collagenases, such as MMP-1 and MMP-13, typically exhibit lower molecular weights around 55 kDa.

**Figure 5:**
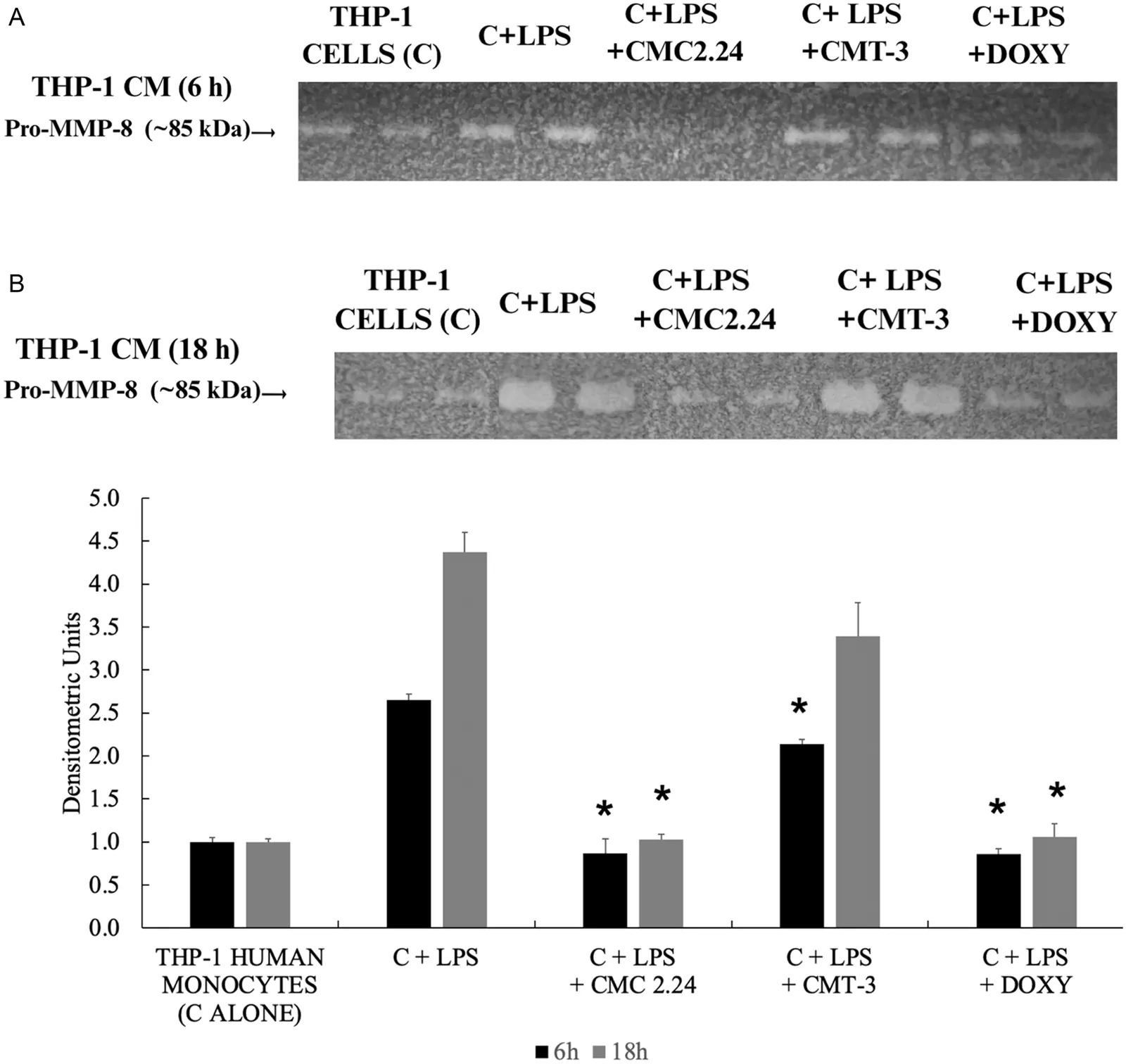
**(A)** Collagenase Levels in THP-1 CM at 6 and 18 h (collagen zymogram). THP-1 cells (1 × 10^6^ cells/ml) were cultured in serum-free media (37°C, 5% CO_2_/95%O_2_) with LPS (50 ng/mL) for 6 or 18 h with 5 µM of CMC 2.24, CMT-3, doxycycline, or vehicle alone. **(B)** Collagenase Levels In THP-1 CM at 6 and 18 H (Quantified by ImageJ). At both 6 and 18 h, CM in Figure 5A were analyzed by collagen zymography for collagenase levels, which were quantified densitometrically using ImageJ. *: *p* < 0.05.

At both 6 and 18 h, the LPS-treated cell CM exhibited significantly higher collagenase levels compared to vehicle alone (Figure 5A). At 6 h, the LPS-treated cell CM increased collagenase levels by 165.35% compared to vehicle alone, and at 18 h, the LPS-treated cell CM increased collagenase levels by 337.29% (Figure 5B).

After 6 h of incubation, collagenase levels were significantly inhibited compared to the cells + LPS control by CMC 2.24 (67.03%, *p* < 0.01), CMT-3 (19.29%, *p* < 0.01), and doxycycline (67.60%, *p* < 0.01).

A one-way ANOVA revealed a significant overall effect of treatment (*p* < 0.001).

After 18 h, collagenases were similarly inhibited, but only by CMC 2.24 and doxycycline (Figure 5). CMC 2.24 significantly reduced collagenase levels by 76.41% (*p* < 0.01), and doxycycline reduced collagenase levels by 76.10% (*p* < 0.01). A one-way ANOVA revealed a significant overall effect of treatment (*p* < 0.001).

### CMC 2.24 reduces collagenolytic MMP enzyme activity

**Figure 6:**
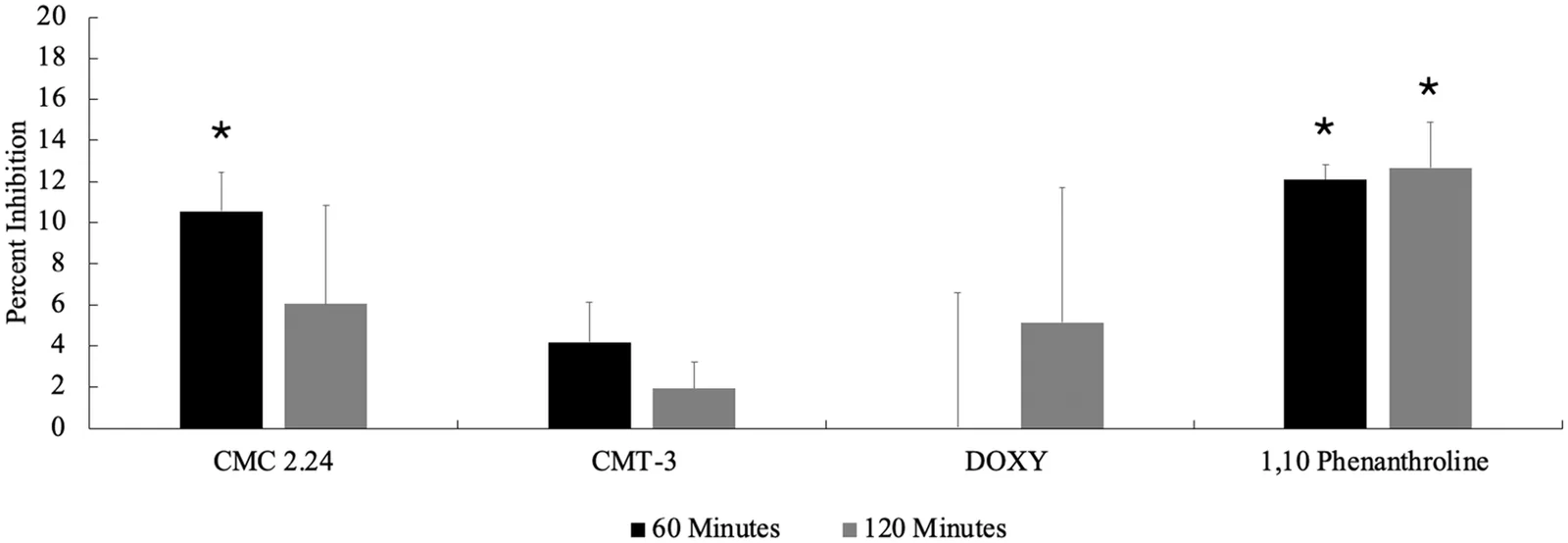
Percent inhibition of collagenolytic MMP activity in LPS-treated THP-1 CM. 18 H conditioned media (CM) of THP-1 cells were incubated with 5 µM of either CMC 2.24, CMT-3, Doxycycline, or 1 mM of 1,10-phenanthroline. Fluorogenic substrate Mca-KPLGL-Dpa-AR-NH₂ was used at 10 µM. Enzyme activity was read at excitation of 320 nm and emission of 405 nm. Activity was determined using a standard curve and relative fluorescence units. Activity was compared, and the percent inhibition for each inhibitor was calculated.*: *p* < 0.05.

Collagenolytic MMP Enzyme Activity was determined using a fluorogenic collagenolytic specific substrate, Mca-KPLGL-Dpa-AR-NH₂. CMC 2.24 reduced collagenolytic MMP enzymatic activity in CM collected from LPS-stimulated THP-1 cells (Figure 6). Specifically, CMC 2.24 decreased collagenolytic MMP activity by 10.5% compared to untreated LPS controls. The one-way ANOVA did not reach statistical significance, (*p* = 0.057). However, a planned pairwise *t*-test identified a significant difference between the CMC 2.24 and LPS control groups (*p* < 0.05). Neither CMT-3 nor doxycycline significantly affected this MMP enzymatic activity in THP-1 CM.

### CMC 2.24 reduces p38 MAPK expression in LPS-stimulated THP-1 cells

**Figure 7:**
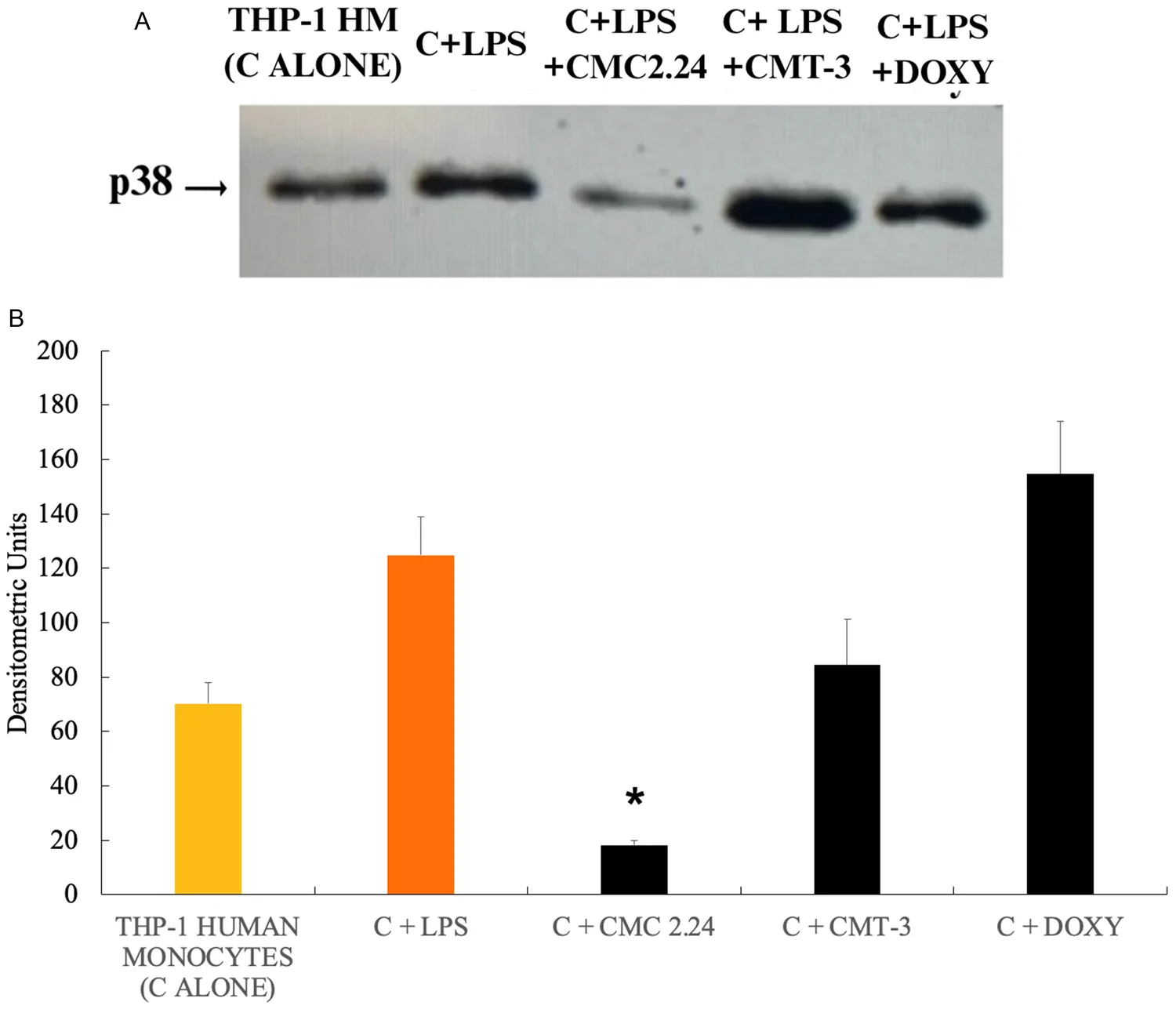
**(A)** p38 levels in THP-1 cells (western blot). THP-1 cells (1 × 10^6^ cells/ml) were cultured in serum-free media (37°C, 5% CO_2_/95%O_2_, 18 h) with LPS (50 ng/mL), CMC 2.24, CMT-3, or Doxy, or vehicle alone. THP-1 cells were centrifuged, lysed, and proteins analyzed via SDS-PAGE and Western blot using p38 MAPK and HRP-conjugated antibodies. **(B)** p38 Levels in THP-1 Cells (Quantified by ImageJ). p38 levels in Figure 7A were detected with SuperSignal™ West Dura, and ImageJ was used to quantify protein levels. Band intensity was normalized via total protein levels and compared. Data are represented as mean ± standard error (SEM). *p < 0.05.

Western blot analysis (Figure 7A) revealed that CMC 2.24 significantly reduced p38 MAPK levels in LPS-stimulated THP-1 cells by an average of 84.17% (*p* < 0.05) compared to LPS-only treated cells (Figure 7B). In contrast, CMT-3 reduced p38 MAPK expression by 22.3%; however, this reduction was not statistically significant. Doxycycline treatment did not result in any significant reduction in p38 MAPK levels. Furthermore, a one-way ANOVA revealed a significant overall effect of treatment (*p* < 0.001).

## Discussion

Periodontal disease is driven by a subgingival biofilm containing Gram-negative pathogens such as *Porphyromonas gingivalis* (*P. gingivalis*), whose outer membrane contains lipopolysaccharide (LPS), a potent endotoxin (1, 2, 20). In periodontal tissues, LPS activates monocytes and macrophages, leading to the production of pro-inflammatory cytokines and MMPs that contribute to connective-tissue destruction and alveolar bone loss (1, 2).

Human monocytic THP-1 cells can be differentiated into macrophage-like cells and therefore model the host response central to periodontal inflammation following LPS stimulation. THP-1 cells were used because they are a well-established human monocytic model that reproduces activation of inflammatory markers present during periodontal inflammation (30–32). Thus, LPS-stimulated THP-1 monocytes provide a periodontally relevant *in vitro* model of the inflammatory response induced by Gram-negative bacteria implicated in periodontitis (1, 2, 20, 30–32).

Previous studies have demonstrated that MMPs play a central role in the degradation of extracellular matrix components and the progression of inflammatory diseases such as periodontitis (1, 2, 21). The present study demonstrates that the chemically modified curcumin analog CMC 2.24 is a pleiotropic inhibitor of matrix metalloproteinase (MMP) levels and activity in LPS-stimulated THP-1 cells. In this context, pleiotropic means that CMC 2.24 does not inhibit only a single MMP or inflammatory mediator, but instead suppresses multiple components of the inflammatory and tissue-destructive response across models. Previous studies from our laboratory have evaluated CMC 2.24 across multiple cell models, including cultured human and rat macrophages, ex-vivo periodontal tissues, and *in-vivo* rat and dog models (1, 2, 21, 35, 36). Across these different models and assays, CMC 2.24 consistently reduced the levels or activity of multiple MMPs and decreased several pro-inflammatory mediators (1, 2, 21, 35, 36).

Previous studies have shown that at CMC 2.24 has no observable toxicity *in-vitro* up to a 25 µM concentration (5 µM and 10 µM used in this study) (37). Additionally, in an *in-vivo* rat study, CMC 2.24 reported no cytotoxic changes in the liver, lungs, kidneys, spleen, colon, or heart at oral doses of 50–1,000 mg/kg/day for five days, or at 100 mg/kg/day for 28 days (35, 36).

After both 6 and 18 h of incubation, LPS increased MMP-9 Levels compared to vehicle alone. At both 6 and 18 h, CMC 2.24 significantly reduced MMP-9 levels, showing greater inhibition than either CMT-3 or doxycycline. These results are consistent with an *in-vivo* study which evaluated CMC2.24 in diabetic rats. In that study, CMC2.24 treatment significantly reduced reduced MMP-9 and inhibited loss of alveolar bone height, volume and mineral density (2).

When assessing the active form of MMP-9, APMA stimulation (16, 35) confirmed that CMC 2.24 reduced the conversion of pro-MMP-9 into its active form more effectively than doxycycline, while CMT-3 showed little to no significant impact. These results suggest that CMC 2.24 can inhibit both expression and activation of MMP-9, targeting multiple points of regulation. It may be possible that CMC 2.24 can prevent the disruption to the cysteine-zinc interaction at MMP-9's N-terminal pro-domain (16, 35), thus reducing activation.

CMC 2.24 also displayed broad inhibitory effects on collagenolytic MMPs (most likely MMP-8), with greater potency than CMT-3 and comparable or superior effects to doxycycline. MMP-1, MMP-8, and MMP-13 are all collagenases capable of degrading type I collagen (8, 9, 12, 13, 17). Based on the molecular weight observed on the collagen zymogram (Figure 4 ∼85 kDa), it is likely that the band represents pro-MMP-8, the most common collagenase in neutrophils and monocytes (8, 17). Western blot analysis would be required to further confirm.

The fluorogenic activity assays also showed mild evidence that CMC 2.24 suppressed overall collagenolytic MMP enzymatic activity. In THP-1 cells, CMC 2.24 inhibited MMP activity at 60 min but not at 120 min, indicating a time-dependent effect. Although the overall one-way ANOVA did not reach statistical significance for MMP enzyme activity in THP-1 cells at 60 min, the planned *t*-test detected a significant difference between the CMC 2.24 and LPS control conditions. This discrepancy reflects the different questions addressed by the two analyses. The ANOVA evaluated whether any overall differences existed across all experimental groups and therefore incorporated variability from every condition, whereas the planned *t*-test focused specifically on the biologically relevant comparison between CMC 2.24 and the Cells + LPS control. Accordingly, the significant pairwise result suggests a condition-specific difference at 60 min, but it should be interpreted cautiously in the context of the nonsignificant ANOVA.

CMC 2.24's broad range of effects can most likely be explained by its phenyl aminocarbonyl structure (1, 21, 37). Due to the metal-ion-binding site capable of interacting with calcium and zinc, CMC 2.24 can inhibit the activity of matrix metalloproteinases which depends on those binding sites. CMC2.24 also contains three phenolic and three ketonic groups, which contribute to its anti-inflammatory, antioxidant, and pleiotropic biological effects (1, 21, 37).

Finally, mechanistic insight was provided by the reduction of p38 MAPK expression in LPS-stimulated THP-1 cells. Activation of the p38 MAPK pathway occurs in response to stress and upregulates the expression of various pro-inflammatory mediators, including MMPs (17, 19). CMC 2.24 suppressed p38 MAPK by over 80%, whereas CMT-3 and doxycycline had limited or no significant effects. This finding is consistent with a previous *in-vivo* beagle dog gingival-tissue study, in which CMC2.24 reduced p38 MAPK expression by 37.2% after three months of treatment compared with the placebo group (*p* < 0.05) (1). The greater reduction observed in the present study may reflect differences in CMC 2.24 administration, exposure duration, experimental model, and tissue complexity. In particular, the present study involved incubation of cultured cells with CMC 2.24 for 18 h, whereas the previous study evaluated the effects of prolonged systemic treatment in heterogeneous gingival tissue. In addition, CMT-3 and doxycycline did not demonstrate significant inhibitory effects on p38 MAPK. It is possible that CMT-3 and doxycycline reduce inflammation through different mechanisms such as the NF-*κ*B pathway (38).

Since p38 MAPK is a central regulator of pro-inflammatory signaling and MMP induction, this pathway may be one of the underlying ways CMC 2.24 acts to reduce MMP levels. It should be noted that GAPDH was not used as a control in this study, as total protein normalization has been shown to offer greater accuracy and reproducibility in Western blot analyses, particularly under inflammatory conditions where housekeeping protein expression may vary. In addition, *β* actin can be used as our internal control for future western blots (39, 40).

Taken together, all these findings indicate that CMC 2.24 is a more effective inhibitor of MMP levels and activation than either CMT-3 or doxycycline. CMC 2.24 may also reduce MMP activity in THP-1 cells. Its ability to target multiple levels of MMP regulation, alongside inhibition of p38 MAPK signaling, highlights CMC 2.24 as a promising candidate for further preclinical development in chronic inflammatory diseases where dysregulated MMP activity contributes to tissue destruction.

The novelty of this study lies in its focus on the *in vitro* effects of CMC 2.24 using undifferentiated THP-1 cells, a model representing early monocytic inflammatory responses. Previous work has primarily demonstrated the efficacy of CMC 2.24 in *in vivo* models, including diabetic rats (35, 36) and beagle dogs with periodontitis (1, 41). By employing an early-stage human monocytic cell line without PMA-induced differentiation, this study provides new evidence that CMC 2.24 suppresses inflammatory signaling and matrix-degrading mechanisms at the cellular level. Furthermore, by using collagen zymography, our findings contribute valuable results on the effect of CMC 2.24 on collagenolytic activity, in addition to their effect on MMP-9.

Importantly, it should be noted that because the primary biological question was whether each treatment reduced MMP-9, MMP-8, active-MMP-9, and p38 levels relative to the stimulated Cells + LPS control, planned independent-samples *t*-tests were used for these specific comparisons. Comparisons among the treatment groups themselves were not part of the prespecified analysis. However, one-way ANOVA was also performed to evaluate whether there was an overall difference across all experimental groups.

Furthermore, since this study is a pilot study, the current findings are based on a limited number of experimental replicates and only the *in-vitro* model. While the results demonstrate consistent and promising inhibitory effects of CMC 2.24 on MMP expression, activation, and activity, future studies will focus on increasing sample size, incorporating additional biological replicates, and expanding to more physiologically relevant models, including differentiated macrophages and *in vivo* systems, to further validate and strengthen these findings. Future directions would also include investigating the effect of CMC 2.24 on additional signaling pathways involved in inflammation, such as NF-*κ*B (42, 43).

## Conclusion

Overall, CMC 2.24 represents a promising therapeutic candidate for modulating inflammation-associated matrix degradation in chronic inflammatory diseases such as periodontitis. The findings of this study demonstrate that CMC 2.24 exerts multiple pleiotropic effects such as inhibiting MMP expression, activation, and activity while also attenuating upstream signaling pathways such as p38 MAPK. Future studies could evaluate the effects of CMC 2.24 in THP-1 cells following PMA-induced differentiation, in order to model macrophage-like inflammatory responses and further validate its therapeutic potential.

## Data Availability

The raw data supporting the conclusions of this article will be made available by the authors, without undue reservation.
